# Supplementary material for: Risk of peripheral artery occlusive disease in patients with lower leg fracture who received fixation and non-fixation treatments: A population cohort study
Source: PLoS One. 2022 Aug 4;17(8):e0272068. doi: 10.1371/journal.pone.0272068 (PMC9352030; doi:10.1371/journal.pone.0272068)
Supplement: Attachment — Submitted filename: 1 st- Response to Reviewers PAOD plos one .docx [file pone.0272068.s001.docx]

**Response to Reviewers’ comments**

PONE-D-22-00911

Risk of Peripheral Artery Occlusive Disease in Patients with Lower Leg Fracture Who Received Fixation and Non-fixation Treatments: A Population Cohort Study

PLOS ONE

Dear Prof. Rudolf Kirchmair and reviewers of PLOS ONE,

Thank you very much for the Letter with the Reviewers’ comments about our manuscript. We greatly appreciate reviewers’ helpful suggestions and the opportunity to revise our manuscript. Efforts have been made to address each comment/concern.

Please find attached revision of the manuscript and see below for our response to the reviewers’ comments.

We look forward to your response.

Yours Sincerely,

Dr. Shih-Chi Wu

Trauma and Emergency Center

China Medical University Hospital

Taichung, Taiwan

Tel: 886-4-22052121 ext 2933

E-mail: rw114@mail.cmuh.org.tw

**Response to Reviewers’ comments**

Reviewer #1: I think it is a very significant paper to improve the quality of trauma care for functional and long-term prognosis. Please correct the following two points.

Major revision

Soft tissue damage and fixation period are thought to influence vessel patency, but if this is the case, it would be better to analyze the fixation methods separately for internal and external fixation.

Response: Thank you very much. We completely agree your comment and greatly appreciate this very crucial point. Yet, owing to database limitation, it would be very difficult to differentiate and analyze the fixation methods separately for internal and external fixation. Therefore, we have added related description in the section of “Limitation”

Also, what was the proportion of minimally invasive plate osteosynthesis among the internal fixations? It would be good to at least mention that in the limitation.

Response: Similar to the above comment. It would be very difficult to measure the proportion of minimally invasive plate osteosynthesis among the internal fixations population. Thank you very much for this point, we have added related description in the section of “Limitation”

Minor revision

In the preface to “Results”, the numerical notation is separated by every three digits, but not thereafter. Please unify either.

Response: Thank you very much, we have made correction.

Reviewer #2:

1. In Table 1, the authors need to include more history of diseases or coexisting medical conditions, such as hyperlipidemia, mental disorders, stroke, Parkinson’s disease, ischemic heart disease, heart failure, COPD, liver cirrhosis, and renal dialysis.

Response: Thank you very much for this point. We greatly appreciate this very crucial comment. We have performed a new analysis following your comment, and added references in the section of “Materials and Methods’. Please refer to the newly revised Table 1-4.

Additionally, we found that there was a significant association between fixation treatment and a reduced risk of lower extremity PAOD in patients with coronary artery disease (Table 3). Therefore, we have added description in the section of “Discussion”

| **Table 1. Demographic characteristics and comorbidities of patients newly diagnosed Fracture of tibia and fibula in Taiwan during 2000-2012** | | | | |
| --- | --- | --- | --- | --- |
| **Characteristics** | **Total** | **Fracture of tibia and fibula** | | **SMD** |
|  |  | **With internal or external fixation N=26152** | **Without internal or external fixation N=6538** |  |
| **Gender** |  |  |  | 0.013 |
| Female | 13683 | 10913 (41.7) | 2770 (42.4) |  |
| Male | 19007 | 15239 (58.3) | 3768 (57.6) |  |
| **Age, mean (SD)** |  | 49.8 (18.3) | 50.0 (18.6) | 0.011 |
| 20-29 | 5911 | 4736 (18.1) | 1175 (18) |  |
| 30-39 | 4680 | 3732 (14.3) | 948 (14.5) |  |
| 40-49 | 5840 | 4644 (17.8) | 1196 (18.3) |  |
| 50-59 | 5904 | 4747 (18.2) | 1157 (17.7) |  |
| 60-69 | 4885 | 3944 (15.1) | 941 (14.4) |  |
| 70-79 | 3974 | 3230 (12.4) | 744 (11.4) |  |
| >=80 | 1496 | 1119 (4.3) | 377 (5.8) |  |
| **Baseline comorbidity** |  |  |  |  |
| Hypertension | 3181 | 2471 (9.4) | 710 (10.9) | 0.047 |
| Diabetes mellitus | 2543 | 1999 (7.6) | 544 (8.3) | 0.025 |
| Gout | 547 | 412 (1.6) | 135 (2.1) | 0.037 |
| Hyperlipidemia | 948 | 721 (2.8) | 227 (3.5) | 0.041 |
| Mental disorder | 648 | 495 (1.9) | 153 (2.3) | 0.031 |
| Stroke | 1271 | 992 (3.8) | 279 (4.3) | 0.024 |
| Parkinson disease | 129 | 106 (0.4) | 23 (0.4) | 0.009 |
| Coronary artery disease | 1474 | 1141 (4.4) | 333 (5.1) | 0.034 |
| Heart failure | 546 | 425 (1.6) | 121 (1.9) | 0.017 |
| COPD | 846 | 663 (2.5) | 183 (2.8) | 0.016 |
| Liver disease | 1617 | 1243 (4.8) | 374 (5.7) | 0.043 |
| ESRD | 170 | 132 (0.5) | 38 (0.6) | 0.010 |
| §A standardized mean difference of ≤0.1 indicates a negligible difference | | | | |

In addition, the use of medical care service also should be considered in this study.

Response: Many thanks for this point. The use of medical care service could be considered equal in Taiwan. Please refer to the following description.

“Taiwan has established the National Health Insurance Program. Inhabitants of Taiwan are obligated to join this health program, which enrolled nearly 99% of residents. Therefore, patients in Taiwan shared almost the same medical care service.

In addition, since 1995, comprehensive health claimed data have been stored in the National Health Insurance Research Database (NHIRD). The NHIRD covered more than 99% of the population in Taiwan. In addition, there were validation studies on this registry which showed that NHIRD is a large, powerful data source for biomedical research.”

*References*:

1. Hsing AW, Ioannidis JP. Nationwide Population Science: Lessons From the Taiwan National Health Insurance Research Database. JAMA Intern Med. 2015 Sep; 175(9):1527-9.
2. Lin LY, Warren-Gash C, Smeeth L, et al. Data resource profile: the National Health Insurance Research Database (NHIRD). Epidemiol Health. 2018;40:e2018062.
3. Hsieh CY, Su CC, Shao SC, et al. Taiwan's National Health Insurance Research Database: past and future. Clin Epidemiol. 2019; 11:349–358.

2. In Table 1, the age should be divided as 20-29, 30-39, 40-49, 50-59, 60-69, 70-79, >=80.

Response: Thanks a lot for this comment. We have performed a new analysis following your comment. Please refer to the newly revised Table 1.

| **Table 1. Demographic characteristics and comorbidities of patients newly diagnosed Fracture of tibia and fibula in Taiwan during 2000-2012** | | | | |
| --- | --- | --- | --- | --- |
| **Characteristics** | **Total** | **Fracture of tibia and fibula** | | **SMD** |
|  |  | **With internal or external fixation N=26152** | **Without internal or external fixation N=6538** |  |
| **Gender** |  |  |  | 0.013 |
| Female | 13683 | 10913 (41.7) | 2770 (42.4) |  |
| Male | 19007 | 15239 (58.3) | 3768 (57.6) |  |
| **Age, mean (SD)** |  | 49.8 (18.3) | 50.0 (18.6) | 0.011 |
| 20-29 | 5911 | 4736 (18.1) | 1175 (18) |  |
| 30-39 | 4680 | 3732 (14.3) | 948 (14.5) |  |
| 40-49 | 5840 | 4644 (17.8) | 1196 (18.3) |  |
| 50-59 | 5904 | 4747 (18.2) | 1157 (17.7) |  |
| 60-69 | 4885 | 3944 (15.1) | 941 (14.4) |  |
| 70-79 | 3974 | 3230 (12.4) | 744 (11.4) |  |
| >=80 | 1496 | 1119 (4.3) | 377 (5.8) |  |
| **Occupation** |  |  |  | 0.013 |
| Office workers | 15109 | 12092 (46.2) | 3017 (46.1) |  |
| Manual workers | 13833 | 11043 (42.2) | 2790 (42.7) |  |
| Others | 3748 | 3017 (11.5) | 731 (11.2) |  |
| **Baseline comorbidity** |  |  |  |  |
| Hypertension | 3181 | 2471 (9.4) | 710 (10.9) | 0.047 |
| Diabetes mellitus | 2543 | 1999 (7.6) | 544 (8.3) | 0.025 |
| Gout | 547 | 412 (1.6) | 135 (2.1) | 0.037 |
| Hyperlipidemia | 948 | 721 (2.8) | 227 (3.5) | 0.041 |
| Mental disorder | 648 | 495 (1.9) | 153 (2.3) | 0.031 |
| Stroke | 1271 | 992 (3.8) | 279 (4.3) | 0.024 |
| Parkinson disease | 129 | 106 (0.4) | 23 (0.4) | 0.009 |
| Coronary artery disease | 1474 | 1141 (4.4) | 333 (5.1) | 0.034 |
| Heart failure | 546 | 425 (1.6) | 121 (1.9) | 0.017 |
| COPD | 846 | 663 (2.5) | 183 (2.8) | 0.016 |
| Liver disease | 1617 | 1243 (4.8) | 374 (5.7) | 0.043 |
| ESRD | 170 | 132 (0.5) | 38 (0.6) | 0.010 |
| §A standardized mean difference of ≤0.1 indicates a negligible difference | | | | |

3. The immortal time bias is very important to this study and the authors need to consider this point in the study design and statistical analysis.

Response: Many thanks for this important comment.

All the patient data in this study came from the population-based hospitalization database, whereas the diagnosis (tibia or fibular fracture) was coded immediately after admission, and the index date initiated in the treatment and non-treatment group. Due to the policy of Diagnosis Related Groups (DRG) in extremity fracture in our National Health Insurance (i.e. package fee for single case with extremity fracture), patients in the treatment group (internal or external fixation) received surgery soon after diagnosis. Therefore, the duration from established diagnosis to treatment was short (within very few days) while the immortal time bias could be neglected.

4. The IRB number seems very old. The authors need to provide the real ethical approval from the Intuitional Review Board.

Response: Thank you very much for this important point.

Please refer to the following IRB certificate.
